# Primary spinal marginal zone lymphoma: an unusual cause of spinal cord compression

**DOI:** 10.11604/pamj.2017.27.171.11947

**Published:** 2017-07-04

**Authors:** Zeineb Alaya, Béchir Achour

**Affiliations:** 1 Department of Rheumatology, Farhat Hached Hospital, Faculty of Medicine of Sousse, Sousse, Tunisia; 2 Department of Hematology, Farhat Hached Hospital, Faculty of Medicine of Sousse, Sousse, Tunisia

**Keywords:** Malt lymphoma, marginal zone lymphoma, spinal tumors, thoracic spine

## Image in medicine

Marginal zone lymphoma (MZL) describes a heterogeneous group of B-cell lymphomas derived from marginal zone cells found in the spleen's white pulp and surrounding germinal centers. To date, only one case of spinal primary MZL has been reported. We report a second case. A 67-year-old man without special medical history was referred to our clinic because of progressive paralysis first noticed 2 months before hospitalization. On examination, his paralysis concerned the lower limbs. No reflex deficits were identified. No further neurological deficits were found. Spinal MRI in sagittal sequence in weighting T2 (A) and sagittal sequence (B); coronal (C) and axial (D) T1-weighted after gadolinium injection showed dorsal compression by an extensive posterior epidural tissue process from T6 to T8 in continuity with left pleural neoplastic thickening through the inter-vertebral homolateral foramens. A laminectomy with resection of the intra-ductal lesion was performed, the examination of which found a MZL. In an extension study, thoraco-abdomino-pelvic CT showed medial and celiac lymphadenopathy, a tissue sleeve of the left costo-vertebral gutter extended to a height of 9 cm. An osteo-medullary biopsy revealed a marrow of normal wealth without sign of malignancy. The lymphoma was classified in bone IV stage. The patient was treated according to the 2013 National Adult Non-Hodgkin's Lymphoma protocol. He received 8 RCHOP cures. The evolution was marked by the improvement of neurological signs with no lesion of evolutive suspicious nature at the thoracic and abdominopelvic stages at the end-of-treatment scan.

**Figure 1:**
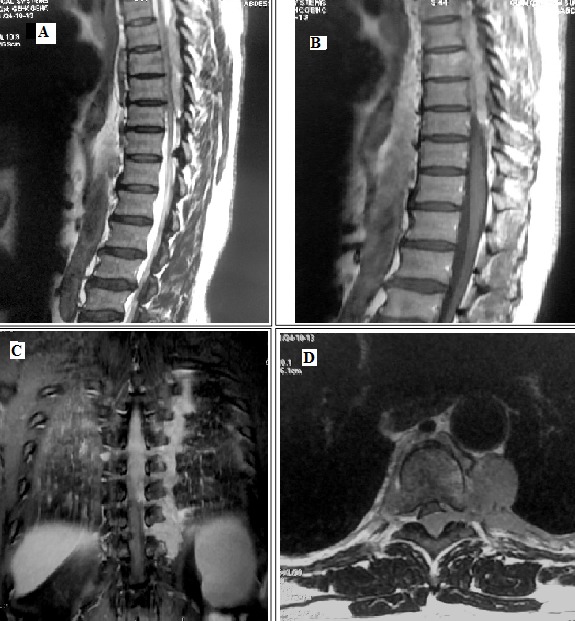
Spinal MRI in sagittal sequence in weighting T2 (A) and sagittal sequence (B); coronal (C) and axial (D) T1-weighted after gadolinium injection showed dorsal compression by an extensive posterior epidural tissue process from T6 to T8 in continuity with left pleural neoplastic thickening through the inter-vertebral homolateral foramens

